# Harmful Cyanobacterial Material Production in the North Han River (South Korea): Genetic Potential and Temperature-Dependent Properties

**DOI:** 10.3390/ijerph15030444

**Published:** 2018-03-03

**Authors:** Keonhee Kim, Chaehong Park, Youngdae Yoon, Soon-Jin Hwang

**Affiliations:** Department of Environmental Health Science, Konkuk University, Seoul 05029, Korea; passbosko@gmail.com (K.K.); qkrcoghd2@gmail.com (C.P.); yyoon21@gmail.com (Y.Y.)

**Keywords:** cyanobacteria, harmful material, geosmin, 2-MIB, microcystin, biosynthesis gene, production potential, environmental hazard

## Abstract

Cyanobacteria synthesize various harmful materials, including off-flavor substances and toxins, that are regarded as potential socio-economic and environmental hazards in freshwater systems, however, their production is still not well understood. In this study, we investigated the potential and properties of harmful materials produced by cyanobacteria, depending on temperature, and undertook a phylogenetic analysis of cyanobacteria present in the North Han River (South Korea). Production potentials were evaluated using gene-specific probes, and the harmful material production properties of strains showing positive potentials were further characterized at different temperatures in the range 15 to 30 °C. We identified six cyanobacterial strains based on 16S rDNA analysis: two morphological types (coiled and straight type) of *Dolichospermum circinale, Aphanizomenon flos-aquae, Oscillatoria limosa, Planktothricoides raciborskii, Pseudanabaena mucicola*, and *Microcystis aeruginosa*. We confirmed that cyanobacterial strains showing harmful material production potential produced the corresponding harmful material, and their production properties varied with temperature. Total harmful material production was maximal at 20~25 °C, a temperature range optimal for cell growth. However, harmful material productivity was highest at 15 °C. These results indicate that the expression of genes related to synthesis of harmful materials can vary depending on environmental conditions, resulting in variable harmful material production, even within the same cyanobacterial strains.

## 1. Introduction

Blooms of cyanobacteria that are known as major producers of diverse harmful materials, including toxins and off-flavor substances, have been reported worldwide, and are regarded as potential hazards, particularly with respect to the maintenance and control of healthy freshwater systems [1,2]. Off-flavor substances, which include geosmin and 2-methylisoborneol (2-MIB), can cause not only tainting of drinking water used for human consumption, but also have negative effects on the marketability of aquaculture products because of their earthy and musty taste and odor. These off-flavor substances are mainly produced by diverse cyanobacteria and *Actinomycetes*, particularly *Dolichospermum circinale* (*Anabaena circinalis*)*, Lyngbya aestuarii, Oscillatoria limosa, Planktothrix agardhii,* and *Pseudanabaena limnetica,* which have been reported as the major cyanobacterial strains in many countries [3,4,5,6]. 

Although water treatment plants employ diverse chemical processes to mitigate off-flavor substance levels [7,8], their removal presents a challenge, given that substances like geosmin and 2-MIB are chemically stable and resistant to oxidation. Moreover, there is a high possibility that harmful by-products will be generated during the chemical treatment processes [9]. In addition to these off-flavor substances, diverse toxins produced by cyanobacteria are also considered as hazardous materials that can threaten the health of both humans and aquatic organisms [10]. Among the diverse cyanotoxins, microcystin is known as a major toxin produced by cyanobacterial strains [11], and is classified into four types, LR, YR, RR, and LA, based on differences in amino acid composition. Although the lethal dose 50 (LD_50_) of microcystin varies from 25 μg/kg to 1000 μg/kg depending on structural type, it is known to be 200 times more toxic than potassium cyanide [11]. Given its acute toxicity, World Health Organization (WHO) guidelines recommend a 1.0 μg/L maximal concentration for drinking water and the water used for irrigation and in leisure complexes [2], and individual countries also set guidelines for the control and maintenance of water quality based on the WHO recommendations [2,10]. 

Secondary metabolites such as off-flavor substances and cyanotoxins are produced within cyanobacterial cells during the exponential phase of growth, and are subsequently released into water systems during the death phase [12]. Hence, these materials tend to be detected only after they have caused their harmful effects. Accordingly, it is necessary to characterize the harmful material production potentials and distributions of harmful cyanobacterial species in freshwater environments in order to provide an early warning system for the control of these cyanobacteria prior to bloom development. 

Traditionally, harmful cyanobacteria have been identified by microscopic observation, and the production of harmful materials has been analyzed chemically using techniques such as high-performance liquid chromatography and gas chromatography. Using these techniques, however, it is difficult to distinguish the morphological variation of cyanobacteria at the strain level caused by diverse environmental conditions [13]. Moreover, the properties of harmful materials produced by different strains of the same species can also vary [14]. In this regard, chromatographic analysis may be insufficient to determine the relationships between specific cyanobacterial strains and the amounts of cyanotoxins in freshwater systems. To overcome the shortcomings associated with the traditional approaches, molecular biological approaches, such as sequence analysis of 16S rDNA and detection of genes involved in harmful material synthesis by PCR, are increasingly being employed to identify cyanobacterial strains and to evaluate harmful material production potentials, respectively [15,16,17,18]. Since genetic variation is generally conservative and occurs more gradually compared with morphological variation [19,20], the phylogenetic analysis of 16S rDNA from cyanobacteria would enable the identification of species regardless of morphological changes. Moreover, production potentials could also be evaluated by probing the genes related to the production of metabolites associated with synthesis pathways of the various toxin and off-flavor substances [18], in addition to providing advance evaluation and prediction of harmful material production potentials [16]. 

The research goals of this study were: (1) to determine the harmful material production potentials of cyanobacteria using selected genes involved in harmful material synthesis, and (2) to evaluate the effect of temperature on the harmful material production of cyanobacterial strains that frequently bloom in the North Han River (South Korea). 

## 2. Materials and Methods

### 2.1. Sampling, Isolation, and Cultivation of Cyanobacterial Strains

**Figure 1:**
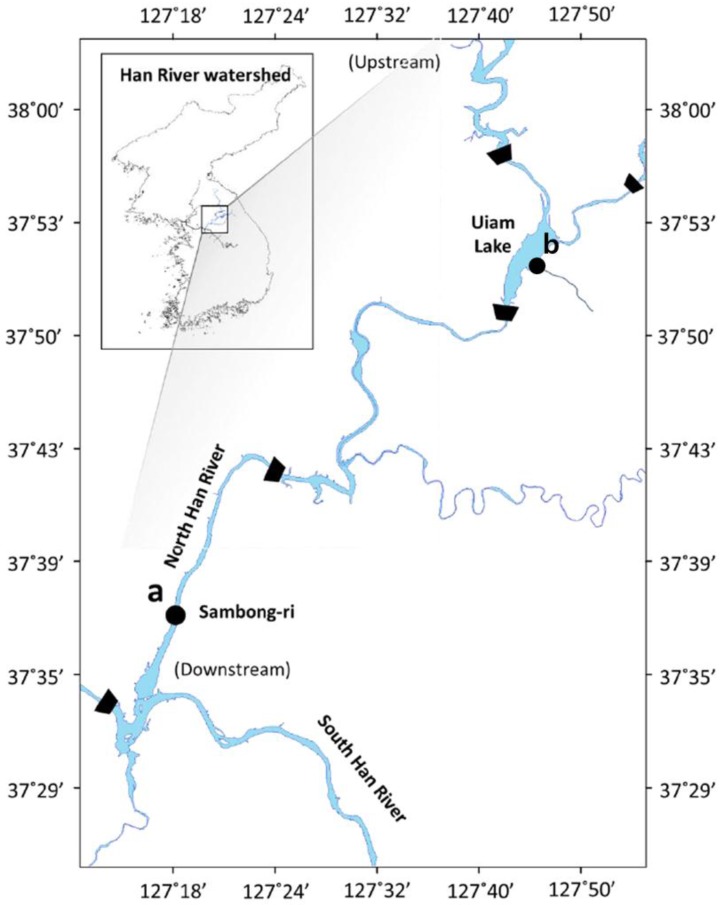
Locations of the North Han River watershed in South Korea and the sampling sites used in this study: (**a**) Downstream of the North Han River; (**b**) Junction with the Kong-ji Stream.

The cyanobacterial strains investigated in this study were isolated from the surface water and sediments sampled from the upstream (37°52′33.8″N, 127°42′22.2″E) and downstream (37°35′41.3″N, 127°20′31.4″E) reaches of the North Han River, South Korea, during July to September in 2015 (Figure 1). The cyanobacterial strains in surface water and sediments were collected by horizontal filtration using a plankton net (pore size: 20 μm, Wildco, Yulee, FL, USA) and with a Peterson Grab sampler (QT Technology, Seoul, Korea), respectively. Sediment was sampled because some cyanobacteria, such as *Oscillatoria* and *Planktothrix*, occur as attached forms. The collected samples were transferred to 100-mL plastic bottles and taken to the laboratory under low temperature (4 °C) conditions. Single strains were isolated from the collected samples using the capillary method [21]. Cells of the single-strain isolates were washed with distilled water and then inoculated into BG-11 medium [22]. The cells were initially grown in 96-well microplates, and then gradually scaled up to 120-mL T-Flasks for further studies. 

### 2.2. Sequencing and Phylogenetic Analysis of the 16S rDNA of Isolated Cyanobacterial Strains

The genomic DNA of isolated cyanobacterial strains was extracted using a Genomic DNA extraction kit (Macherey-Nagel, Valencienner Straße, Düren, Germany) following the protocol provided by the manufacturer. Briefly, the cells were lysed by vortexing in lysis solution containing sodium dodecyl sulfate and silica beads, and a supernatant was obtained by centrifugation (1730R; LOBOGENE, Seoul, Korea). The supernatant was applied to a micro-column to isolate the genomic DNA. The genomic DNA of each strain was used as a template for polymerase chain reaction (PCR) analysis. The sequences of the primers used for PCR amplification are listed in Table 1. The 16S rDNA sequences were then analyzed by DNA sequencing using an ABI 3730XL DNA analyzer (Perkin-Elmer, Waltham, MA, USA). The 16S rDNA sequences of the cyanobacterial strains thus obtained were used for BLAST searches of the GenBank database of the National Center for Biotechnology Information (NCBI, Bethesda, MD, USA). The sequence of each strain was aligned using the ClustalW algorithm, and each strain was identified based on the hit score from the BLAST search.

For phylogenetic analysis, the sequences of 16S rDNA from diverse cyanobacteria, including *Microcystis, Aphanizomenon, Dolichospermum* (*Anabaena*)*, Oscillatoria, Planktothricoides* (*Planktothrix*), and *Pseudanabaena*, were obtained from NCBI. Alignment of the 16S rDNA sequences obtained from isolated strains and the database was performed using ClustalW. The phylogenetic analysis was then performed based on the maximum-likelihood method using MEGA 6.0 (NIH, Bethesda, MD, USA) [15] with 1000 bootstrap iterations to verify the phylogenic positions. 

### 2.3. Evaluation of Harmful Material Production Potentials

Harmful material production potential was evaluated by determining the presence of genes related to the synthesis of these harmful materials, namely, microcystin, geosmin, and 2-MIB, by PCR assays. In cyanobacteria, microcystin, geosmin, and 2-MIB are synthesized via the activities of a series of genes, and the harmful material production potential can be assessed by measuring the levels of certain target genes, such as *mcyA*, *gys,* and *mibC*, that encode core enzymes involved in the synthesis of microcystin, geosmin, and 2-MIB, respectively. To detect the presence of these genes, we employed PCR assays using target gene-specific primers. In the case of *gys* and *mcyA*, we used primers reported in previous studies [16,26], whereas the primers used to detect 2-MIB were designed based on the *mibC* sequences of *Pseudanabaena, Oscillatoria, Planktothricoides* which were registered in GenBank (accessions: HQ630887.1, HQ630883.1, HQ830028.1, HQ630885.1, KJ658378, KJ658377.1, KM013396.1, LC157992.1). Information on the three sets of primers is listed in Table 1. The PCR products were analyzed by agarose gel electrophoresis and sequenced for further verification. The predicted sizes of the PCR products for *gys*, *mibC,* and *mcyA* genes were 565, 196, and 291 bp, respectively. 

### 2.4. Analysis of Harmful Materials Produced by the Isolated Cyanobacterial Strains 

In order to determine the amount of off-flavor substances produced (geosmin and 2-MIB), the cyanobacterial samples were treated using the head space-solid phase micro-extraction method (HS-SPME). The samples were activated at 270 °C for 1 h with helium gas, and then adsorbed onto SPME fiber for 30 min with 3 g of NaCl. The samples were desorbed for 4 min at 270 °C before analyzing by gas chromatography/mass spectrometry (GC/MS) (450-GC, 320-MS; Bruker, Billerica, MA, USA) [27]. In the case of microcystin, the cyanobacterial samples were sonicated for 30 min (ULH-7000s; Jeiotech, Seoul, Korea). The lysed cells were filtered using CF/C filters, and then the microcystin was separated using a solid-phase cartridge. Microcystin was analyzed by liquid chromatography/mass spectrometry (LC/MS) (LC 1260 Infinity/320-MS, Agilent Technologies, Lexington, MA, USA) using an ODS C_18_ column (4.6 mm × 150 mm, 5 μm) [27]. 

### 2.5. Analysis of Temperature Effects on Harmful Material Production 

To elucidate the effect of temperature on the production of harmful materials by cyanobacteria, isolated cyanobacterial strains that have been determined to produce off-flavor substances and toxins were grown at different temperatures ranging from 15 °C to 30 °C (15, 20, 25, and 30 °C). Cyanobacteria isolated from the North Han River were used to inoculate 100 mL of BG-11 medium in 250-mL flasks and grown to a final concentration of 10^4^ cells/mL. The cyanobacterial strains were grown in a shaking incubator (VS-1203P4S; VISION, Daejon, Korea) for 14 days. The growth of all cyanobacterial strains were reached to the stationary phase after 11 days of incubation. Then the concentrations of chlorophyll-*a* (Chl-*a*) and harmful materials (geosmin, 2-MIB, and total microcystin) in 10-mL aliquots were analyzed by following standard methods [28] and by using procedures described above, respectively. All analyses were performed using three replicates. For comparisons of the effects of temperature on the production of harmful materials by cyanobacteria, productivity was expressed as (total amount of harmful materials)/(the amount of biomass (Chl-*a*)).

### 2.6. Registration of Isolated Cyanobacterial Strains (Accession Numbers)

In this study, we isolated seven cyanobacterial strains from the North Han River and investigated their morphological and molecular characteristics. The 16S rDNA sequences of these seven strains and the phylogenetic analysis data have been registered in the DNA Database Bank of Japan (DDBJ). The accession numbers of the seven isolated strains are as follows: LC006113 for *Dolichospermum circinale* (*Anabaena circinalis*) straight-type, LC006112 for *D. circinale* coiled-type, LC178835 for *Aphanizomenon flos-aquae*, LC178836 for *Microcystis aeruginosa*, LC178838 for *Oscillatoria limosa*, LC178839 for *Planktothricoides raciborskii,* and LC177664 for *Pseudanabaena mucicola*.

## 3. Results

### 3.1. Phylogeny of the Isolated Cyanobacterial Strains

**Figure 2:**
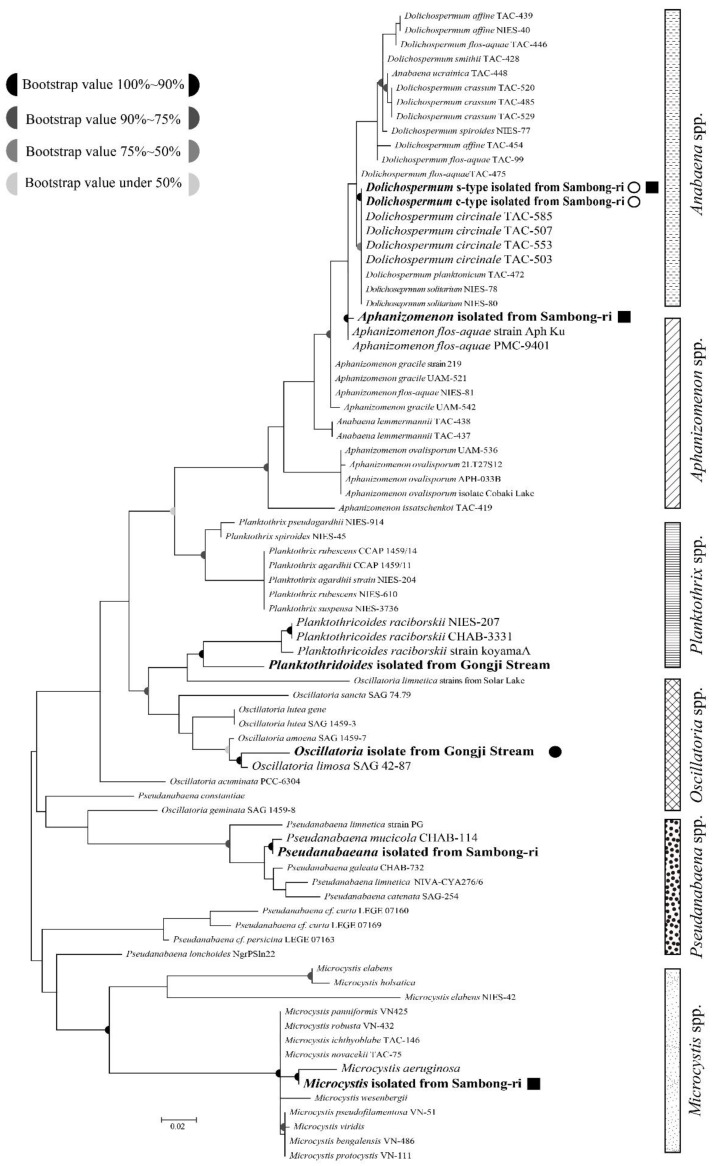
Phylogenic relationships of Nostocales, Oscillatoriales, Synechococcales, Chroococcales taxa. White circles indicate the cyanobacterial strains isolated from the North Han River. The former name of the genus *Dolichospermum* is *Anabaena*. Open circles: geosmin-producing cyanobacteria, black circles: 2-MIB-producing cyanobacteria, black squares: microcystin-producing cyanobacteria.

The phylogeny of the 16S rDNA sequences of seven cyanobacterial strains isolated from the North Han River were analyzed by comparing with the 16S rDNA sequences from 81 strains of six species detected in the NCBI database. 16S rDNA sequences comprising 1306 nucleotides were compared, and we found that the conserved regions in all strains consisted of 743 nucleotides (56.8%) and that homology between all strains ranged from 50% to 60%. The parsimony-informative region, the criterion used for the phylogenetic classification, consisted of 371 nucleotides (28.4%). Accordingly, the phylogenetic classification between cyanobacterial strains was determined based on differences in less than 30% of the analyzed 16S rDNA sequence. The 16S rDNA amplified from the genomic DNA of each of the isolated strains was sequenced, and the strains were accordingly identified as *Dolichospermum circinale* (*Anabaena circinalis*)*, Aphanizomenon flos-aquae, Oscillatoria limosa, Planktothricoides raciborskii, Pseudanabaena mucicola,* and *Microcystis aeruginosa* (Figure 2). 

### 3.2. Harmful Material Production Potentials

**Figure 3:**
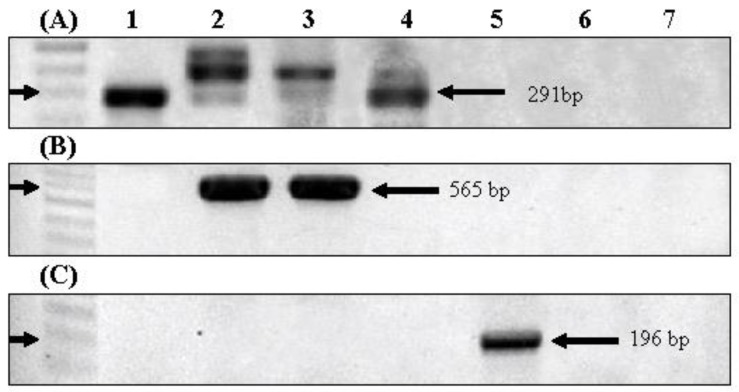
Agarose gel electrophoresis of PCR amplicons for (**A**) microcystin (*mcyA* gene), (**B**) geosmin (*gys* gene), and (**C**) 2-MIB (*mibC* gene) synthesis genes. 1: *Microcystis aeruginosa*, 2: *Dolichospermum circinale* (*Anabaena circinalis*) ST, 3: *D. circinale* (*A. circinalis*) CT, 4: *Aphanizomenon flos-aquae*, 5: *Oscillatoria limosa*, 6: *Planktothricoides raciborskii*, 7: *Pseudanabaena mucicola*.

As shown in Figure 3A, 291-bp PCR products were amplified from *M. aeruginosa*, *D. circinale*, and *A. flos-aquae* using *mcyA*-targeted primers. In the case of *D. circinale*, a band of the expected size was clearly detected only from the straight-type, whereas the coiled-type showed a smeared band. The PCR products were further sequenced to confirm their identity, and were accordingly identified as *mcyA* genes, with the exception of the PCR product obtained from coiled-type *D. circinale*. Furthermore, among the seven isolated strains, *gys,* which is indicative of geosmin production potential, was detected from both straight and coiled types of *D. circinale*, and *mibC* was detected from *O. limosa* (Figure 3B,C). 

The cyanobacterial species isolated in the North Han River have previously been reported in many countries, and are known producers of odorous substances and microcystin (Table 2). In the case of *M. aeruginosa* and *P. mucicola*, the production of off-flavor substances has not been previously reported from any country. Consistently, none of the genes associated with the synthesis of odorous substances were detected from the *M. aeruginosa* and *P*. *mucicola* isolated in the present study. Additionally, to date, there have been no reports of the production of microcystin by *P*. *mucicola*, and no amplicons of the *mcyA* gene were detected in our PCR assay. However, the production of microcystin by *P*. *mucicola* has previously been reported in Morocco (Table 2). Although *P. raciborskii* has been reported to produce microcystin and off-flavor substances in the USA and China, no amplicons of *mcyA* were detected from the *P. raciborskii* isolated in the present study, which is consistent with the fact that there have been no previous reports of microcystin production by *P. raciborskii* in South Korea. 

On the basis of our PCR analyses, we propose that the *M. aeruginosa*, *D. circinale,* and *A. flos-aquae* isolated in this study possess microcystin production potential, and that *D. circinale* and *O. limosa* are geosmin and 2-MIB producers, respectively. Additionally, neither *P. mucicola* nor *P. raciborskii* are responsible for the production of microcystin or off-flavor substances in the North Han River. On the basis of our BLAST search results (Supplementary Material Table S1), it was revealed that the sequences of *mcyA* detected from *M. aeruginosa* and *A. flos-aquae* show over 98% homology with the microcystin synthase (*mcyA*) gene of *M. aeruginosa* FCY-26 (JQ290083.1) isolated from Lake Paldang located in the upstream region of the North Han River. The *mcyA* from *D. circinale* straight-type showed 79% sequence homology with the *mcyA* gene of *Dolichospermum* (*Anabaena*) sp. 0tu33s16 identified from Lake Tuusulanjärvi in Finland. The sequences of *gys* from both types of *D. circinale* and that of *mibC* from *O. limosa* showed over 95% homology with the geosmin synthase gene (HQ404997.1) of the *A. ucrainica* CHAB strain isolated from Lake Dianchi in China and the MIB synthase gene (HQ630885.1) of *O. limosa* LBD 305b strain from the Center for Inland Water in Canada, respectively. 

### 3.3. Temperature Dependency of Harmful Material Production by the Isolated Cyanobacteria

**Figure 4:**
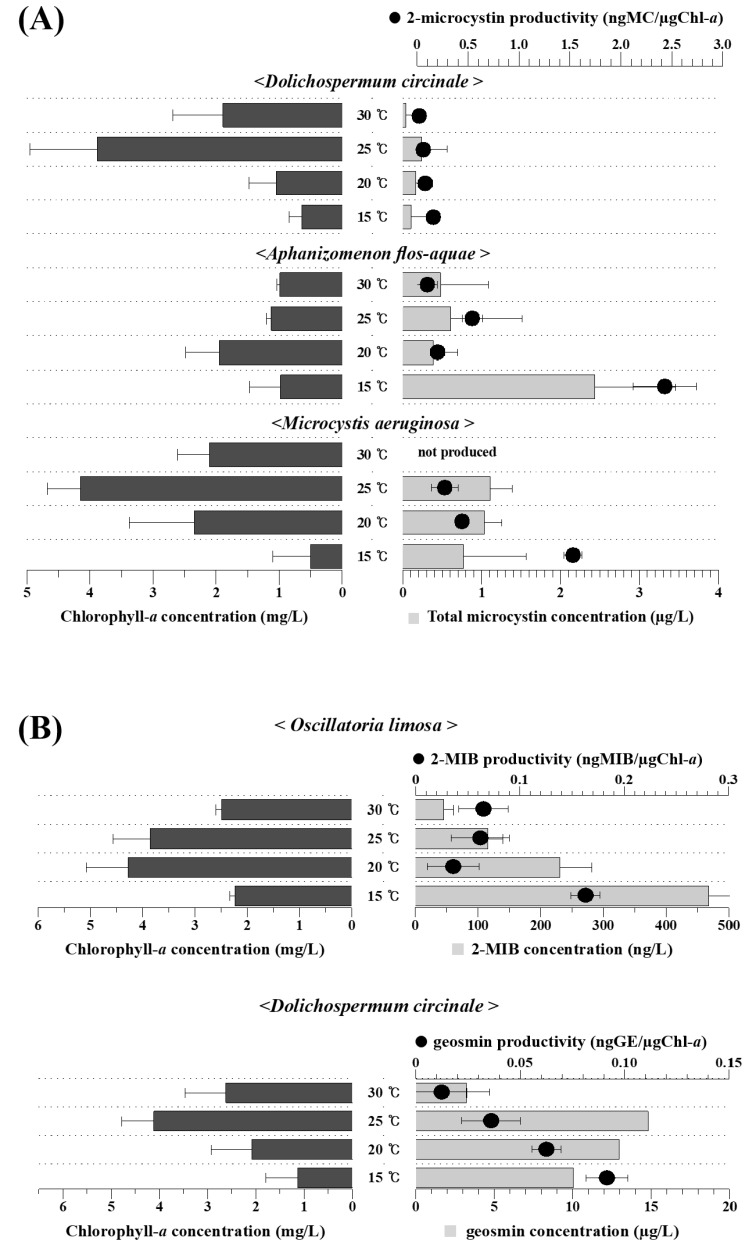
Concentration of chlorophyll-*a* (black bars) and the harmful materials (gray bars) microcystin (**A**) and geosminand 2-MIB (**B**) produced by various cyanobacteria at different temperatures. Black circles indicate productivity (the ratio of harmful material concentration to chlorophyll-*a*concentration) of harmful material produced by cyanobacteria.

In order to confirm the harmful material production potentials of the cyanobacteria evaluated by PCR analysis, we determined the amounts of microcystin, geosmin, and 2-MIB produced by the cyanobacterial isolates (Figure 4). Microcystin and off-flavor substances (geosmin and 2-MIB) were detected from cyanobacterial strains expressing the harmful material synthesizing genes, namely, *M. aeruginosa*, *D. circinale*, *A. flos-aquae,* and *O. limosa*. However, no harmful material was detected from *P. mucicola* and *P. raciborskii,* for which no related amplicons were detected by PCR assay. 

Among the four cyanobacteria possessing harmful material production potentials, *O. limosa* had the highest biomass (Chl-*a* concentration) of 3.21 mg/L during 14 days of incubation, whereas the Chl-*a* concentrations *of D. circinale* and *M. aeruginosa* were 2.37 mg/L and 2.01 mg/L, respectively (Figure 4). Under the experimental conditions, *A. flos-aquae* produced the lowest biomass (1.44 mg/L). It was observed that the growth rate of cyanobacteria varied according to incubation temperature. Maximum biomasses of *D. circinale* and *M. aeruginosa* were produced at 25 °C, whereas those of *A. flos-aquae* and *O. limosa* were obtained at 20 °C. However, all the examined cyanobacteria showed slowest growth at 15 °C, whereas cell death occurred at 30 °C, resulting in an approximate 40% decrease in biomass. 

Although we found that the harmful material production of cyanobacteria varied according to temperature, the temperatures for maximum biomass and harmful material production did not always coincide. Whereas the harmful material concentration and biomass of *Dolichospermum* and *Microcystis* were maximized at the same temperature, 25 °C, we found that *Aphanizomenon* and *Oscillatoria* produced the highest concentration of microcystin and 2-MIB, respectively, at 15 °C, a temperature at which the concentration of chlorophyll-*a* was lowest among those examined (Figure 4). Nonetheless, it was noticed that harmful material productivity was increased at 15 °C when the growth rate of cyanobacteria was decreased. In the case of microcystin, *A. flos-aquae* showed highest productivity (2.73 ng/μg Chl-*a*) at 15 °C, followed by *M. aeruginosa* and *D. circinale*, with productivities of 1.59 and 0.16 ng/μg Chl-*a*, respectively (Figure 4A). Although the amounts of 2-MIB and geosmin were relatively lower than those of microcystin, the geosmin productivity of *D. circinale* (0.06 × 10^3^ ng/μg Chl-*a*) was 1500 times higher than the 2-MIB productivity of *O. limosa* at 15 °C (0.04 ng/μg Chl-*a*). However, it was comparable to the harmful material productivity determined in previous studies at the same temperature. As listed in Table 3, the average microcystin productivity of the *M. aeruginosa* investigated in the present study was approximately three times higher than the recorded from *Microcystis* isolated in France, Canada, and Japan, whereas *D. circinale* and *A. flos-aquae* showed 13 and 1.8 times lower values than strains isolated from Japan and France, respectively. The productivity of the off-flavor substances geosmin and 2-MIB recorded in the present study was not significantly different from that of other strains isolated in South Korea and other countries (Table 3).

## 4. Discussion

Blooms of microcystin-producing cyanobacteria, such as *Microcystis*, *Dolichospermum* (*Anabaena*), and *Aphanizomenon*, occur worldwide, causing diverse problems, particularly with respect to drinking water sources [49,50]. Major producers of off-flavor substances, such as *Dolichospermum* and *Oscillatoria*, are also widely distributed and known to cause problems in water-related industries [51]. Our study showed that these cyanobacteria possess corresponding harmful material production potentials in the North Han River, as demonstrated by the presence of the biosynthesis genes *mcyA*, *gys,* and *mibC,* which encode core enzymes that participate in the synthesis of microcystin, geosmin, and 2-MIB, respectively. The detection of such genes can provide an early warning indicator of cyanobacterial harmful material production in situ. Furthermore, we suggest that molecular biological approaches need to be employed for the control and maintenance of freshwater quality based on a better understanding of ecological dynamics, such as those pertaining to blooms and harmful material production potentials in cyanobacterial communities. 

In the case of *Dolichospermum* (*Anabaena*) strains, although their shapes are obviously different, both coiled and straight types were classified are the same taxon, the *D. circinale* TAC strain isolated from Japan, with 95% bootstrap support. Although *D. circinale* isolated from the North Han River clustered in the same phylogenetic group as *Dolichospermum planktonicum* (*Anabaena planctonica*) and *Dolichospermum solitarium* (*Anabaena solitaria*), there were variation in the shapes and sizes of cells and akinetes [13]. Moreover, the shapes of the trichomes of *Dolichospermum* cells could be distinguished as two different types, straight-type (*D. circinale* straight-type and *D. planktonicum*) and coiled-type (*D. circinale* coiled-type and *D. solitarium*), even though they were clustered in the same phylogenetic group. Conversely, *D. circinale* showed similar morphological characteristics, such as shapes and sizes of cells, trichomes, and akinetes, to *Dolichospermum crassum* (*Anabaena crassa*) [13,52,53], even though they clustered into different groups in the phylogenetic tree. Such discrepancies tend to occur because taxonomic systems based on morphological characteristics of cyanobacterial strains are often not representative of differences at the molecular level. A similar discrepancy between morphological and genetic taxonomic systems has also been observed at the genus level for *Microcystis* in this study. In spite of morphological differences, such as size of cell and ectoplasm membrane [54,55], diverse species of *Microcystis* are classified into the same phylogenetic groups with greater than 90% bootstrap support, notably *M. panniformis, M. robusta, M. ichthyoblabe*, and *M. novacekii* that show 98% 16S rDNA sequences similarity, and *M. pseudofilamentosa, M. viridis, M. bengalensis,* and *M. protocystis* that show 99% similarity.

We observed that the harmful material production capability of cyanobacteria is temperature dependent, suggesting that differences in environmental conditions and the geographical distribution among cyanobacteria have effects on the function of genes associated with cyanotoxin and off-flavor substance production. For example, *D. circinale* and *D. flos-aquae* isolated from Australia and Brazil produce saxitoxin [2,56], whereas *D. circinale* from the USA produces only anatoxin-a [56]. Additionally, the same species of *D. circinale* from France produces microcystin [29], whereas no cyanotoxin has been detected from this species in Japan [57]. Accordingly, it is not possible to evaluate the production of harmful materials by cyanobacteria based solely on taxonomic classification. In general, several genes related to synthesis pathways for harmful materials exist as clusters (operons), and the order of genes in such clusters may vary among different species of cyanobacteria [58]. The genes involved in the biosynthesis of microcystin comprise a cluster of 10 genes, named *mcyA* to *mcyJ,* and each gene contributes to generate the chemical structure of the final product [59]. In the case of off-flavor substances, clusters comprising three and four genes, including the putative geosmin synthase (*gys*) and monoterpene cyclase (*mibC*) genes, are key genes in the synthesis of geosmin and 2-MIB, respectively [16,60]. Therefore, the harmful production potentials of cyanobacteria could be evaluated by measuring the presence of these genes using a simple PCR assay [61]. Indeed, the genes *mcyA* and *mcyE* have already been used as molecular markers to evaluate microcystin synthesis potential in the field, as have the *gys* and *mibC* genes associated with geosmin and 2-MIB synthesis, respectively [25,62]. However, the presence of these genes does not invariably coincide with the production of harmful materials. Indeed, the amounts of harmful materials produced by cyanobacteria vary depending upon physicochemical environmental conditions, and under certain circumstances no harmful materials are produced [63]. Moreover, the function of genes that participate in the production of harmful materials may change when mutations are introduced into these genes [64]. In this regard, it cannot be categorically asserted that the detection of genes involved in the synthesis of cyanobacterial harmful materials in the field invariably coincides with the actual production of these harmful materials. Therefore, it would be necessary to employ the instrumental analysis for determining the amounts of harmful materials as well as genetic analysis for evaluating the harmful material production potentials. 

The cyanobacteria investigated in the present study showed maximal production of harmful materials in the temperature range 20–25 °C when the biomass (Chl-*a*) production was maximal, whereas harmful material productivity (the amount of materials per biomass unit) was highest at 15 °C when the biomass was lowest. This pattern of productivity might be explained by the fact that the cyanobacteria are more likely to produce secondary metabolites than to increase biomass when they encounter unfavorable growth condition [65,66]. On the basis of this assumption, it can be inferred that they produce more harmful materials at low temperature and low light intensity [16,67], particularly in the case of the odor and taste substances that are synthesized from the isoprenoid pathway, which is the same route by which cyanobacterial chlorophyll-*a* is synthesized [16]. Unfavorable growth conditions might therefore lead to an increase in geosmin and 2-MIB synthesis relative to chlorophyll-*a,* resulting in the accumulation of geosmin and 2-MIB in cells. This finding has significance from both ecological and environmental perspectives. Under unfavorable conditions (e.g., low temperature and low light), the accumulation of biomass by cyanobacteria via the synthesis of chlorophyll-*a* would not be an energetically efficient survival strategy, and accordingly they could switch from chlorophyll-*a* to secondary metabolite synthesis [16]. Indeed, low temperature and low light are limiting factors for cyanobacterial growth and photosynthesis [68]. The occurrence of cyanobacteria that produce harmful materials during the cold season, by virtue of being unexpected, may necessitate an extra effort to control. Such an outcome was reported in a drinking water resource located in the North-Han River (Korea) in December 2011 when the water temperature was approximately 5 °C [69]. 

Comparing the harmful material production capacity of the cyanobacteria isolated in the present study with that of cyanobacteria in other freshwater systems, we found that microcystin productivity was quite variable, whereas the production of off-flavor substances (geosmin and 2-MIB) tended to be more similar. In addition, it was noticed that the temperatures for optimum growth and maximum harmful material productivity reported from other countries were higher than those recorded for the North Han River [70]. This difference could be explained by the fact that the environmental conditions experienced by cyanobacteria strains has an effect on the growth and the activity of harmful material synthesizing genes, as has been indicated in previous studies [2,29,71]. For example, *Microcystis* isolated from the North Han River enter the death phase and produce less cyanotoxins at 30 °C, whereas *Microcystis* from other countries, such as the USA, Australia, and China, produce more cyanotoxins at the same temperature [70,72]. High temperatures in excess of 30 °C in our study area, the North Han River, were recorded in 8 years (25 times from 547 samplings) during the 27-year period between 1990 and 2016 (Water Information System: water.nier.go.kr). Furthermore, the temperatures ranging between 20 °C and 25 °C were recoded every year (156 times), and temperature of 25 ± 0.5 °C were measured 26 times during a span of 15 years. The average concentration of Chl-*a* measured at temperatures above 30 °C was 18.4 μg/L, which is approximately two times lower than the 38.4 μg/L recorded at 25 (±0.5) °C. Moreover, *Microcystis, Dolichospermum,* and *Aphanizomenon* isolated from the study area showed the highest Chl-*a* concentrations at 25 °C. Hence, the temperature for maximum growth rate and harmful material productivity of cyanobacteria strains occurring in the North Han River differ from those occurring in other countries. Therefore, it can be inferred that the geographical distribution and environmental conditions of cyanobacterial habitats have an influence on the expression of genes involved in harmful material biosynthesis.

To date, there have been no reports regarding 2-MIB production by *O. limosa* isolated in South Korea. In the case of microcystin, production by *Microcystis*, *Dolichospermum,* and *Aphanizomenon* was reported during the period from 2005 to 2009, and the amounts of geosmin produced have previously been shown to increase explosively coinciding with the blooms of *Dolichospermum*, particularly in 2012 [69,73]. However, these results do not provide information on the direct relationship between the occurrence of cyanobacterial species and their harmful material production because they have been based on statistical relationships between the major species and the concentration of harmful materials. Accordingly, it is necessary to elucidate the causal relationships between cyanobacteria species and harmful materials. In this regard, the results of the present study go some way to verifying the relationship between the harmful material production potentials and actual harmful material production of the isolated cyanobacterial strains based on molecular biological approaches and instrumental chemical analyses, respectively. 

## 5. Conclusions

In this study, we evaluated the genetic potential and temperature-dependent properties of harmful material production by cyanobacteria occurring in the North Han River (Korea). We revealed that the major cyanobacteria produce certain specific harmful materials (microcystin, geosmin, and 2-MIB), and that production potential was associated with the presence of genes related to the biosynthesis of these materials. We also observed that the production of these materials varied depending on cyanobacterial species and growth conditions, and that the expression of genes related to harmful material production was affected by environmental conditions. 

We believe that our findings are very meaningful from an environmental perspective, in that the capacity of cyanobacteria to produce harmful materials was increased under unfavorable growth conditions (i.e., low temperature). These results emphasize that we need to show awareness regarding cyanobacterial occurrence in low-temperature seasons, i.e., late fall and winter, in addition to the blooming season when they develop maximal biomass. Indeed, the need for such awareness is well illustrated by an episodic event in our study region, when geosmin concentration hit a record high during December of 2011.

On the basis of our study results, we suggest that a phylogenetic analysis would be necessary to identify cyanobacterial strains that show similar morphological characteristics such as sizes and shapes of cells at the species and genus levels. Our results could provide information regarding the relationship between the genetic variation of strains and their harmful material productivity dependent upon differences in geographical distribution and environmental conditions of the harmful material-producing cyanobacteria. Finally, we suggest that molecular biological methods represent an alternative approach for evaluating the harmful production potentials associated with the blooms of cyanobacteria, thereby providing an early warning of the need for measures to control the blooms of harmful cyanobacteria and maintain healthy water quality.

## Figures and Tables

**Table 1:** Information on the primers used in this study.

Primer	Direction	Sequence (5′→ 3′)	Expected Size	Melting Temp	Reference
16S rDNA	Forward	GGGGAATTTTCCGCAATGGG	1284 bp	60 ℃	[23]
	Reverse	ACCTTGTTACGACTT			
geosmin (*gys1*)	Forward	CTA GAC CMA TGC GGG TTT TA	569 bp	56 ℃	[16]
	Reverse	CCA TTC TTT RGA ATG MTT			
2-MIB (*mibC*)	Forward	ACG ACA GCT TCT ACA CCT CCA TGA	196 bp	62 ℃	[24]
	Reverse	AAT CTG TAGCAC CAT GTT GAC WGG TG			
microcystin (*mcyA*)	Forward	AAA AGT GTT TTA GCG GCT CAT	291 bp	54 ℃	[25]
	Reverse	AAA ATT AAA AGC CGT ATC AAA			

Mixed oligo: R(A,G) Y(C,T) M(A,C) K(G,T) S(C,G) W(A,T) H(A,C,T) B(C,G,T) V(A,C,G) D(A,G,T) N(A,C,G,T).

**Table 2:** Comparison of harmful cyanobacteria and the types harmful material produced in different geographic locations.

Cyanobacteria Species	Cyanotoxin	Location	Reference	Off-Flavor Compound	Location	Reference
*Dolichospermum circinale* (*Anabaena circinalis*)	microcystin, anatoxin-a saxitoxin	France Finland USA S. Korea	[29,30,31] and This study	geosmin	Australia S. Korea	[6,13,16] and This study
*Aphanizomenon flos-aquae*	microcystin, anatoxin-a saxitoxin	USA Canada New- Zealand S. Korea	[30,32,33] and This study	geosmin	USA	[34]
*Microcystis aeruginosa*	microcystin	Canada Japan S. Korea	[35,36,37,38] and This study	Not reported	-	Not reported
*Planktothricoides raciborskii*	microcystin	USA	[39]	2-MIB	China	[40]
*Oscillatoria limosa*	microcystin	Switzerland	[41]	geosmin, 2-MIB	USA Spain S. Korea	[4,42,43] and This study
*Pseudanabaena mucicola*	microcystin	Morocco	[44]	Not reported	-	Not reported

**Table 3:** Comparison of productivities of microcystin and off-flavor materials (geosmin and 2-MIB) produced by various cyanobacteria. Values were averaged from the same temperature range as for the reference cyanobacteria.

Microcystin and Off-Flavor Materials	*Microcystis*	*Dolichospermum* (*Anabaena*)	*Aphanizomenon*	*Oscillatoria*	Reference
**Microcystin**	0.12 (±0.08) ngMC/μgChl-*a*	0.26 (±0.11) ngMC/μgChl-*a*	0.68 (±0.54) ngMC/μgChl-*a*	0.02 (±0.01) ngMC/μgChl-*a*	Shown underneath each material value
	[36,37,45]	[29,35,46]	[46]	[47]	
	0.37 (±0.15) ngMC/μgChl-*a*	0.02 (±0.01) ngMC/μgChl-*a*	0.38 (±0.26) ngMC/μgChl-*a*	-	This study
**Odorous materials**	-	0.05 (±0.05) × 10^3^ ngGE/μgChl-*a*	-	0.03 (±0.01) ngMIB/μgChl-*a*	Shown underneath each material value
		[48]		[40,48]	
	-	0.06 (±0.02) × 10^3^ ngGE/μgChl-*a*	-	0.04 (±0.02) ngMIB/μgChl-*a*	This study

Remarks; MC: Total microcystin, GE: geosmin, MIB: 2-methylisoborneol (2-MIB).

## Supplementary Materials

The following are available online at http://www.mdpi.com/1660-4601/15/3/444/s1, Table S1: BLAST result of harmful-material-synthesizing genes of cyanobacterial strains isolated from the North-Han River, S. Korea.

## References

[1] Olsen B.K., Chislock M.F., Wilson A.E. (2016). Eutrophication mediates a common off-flavor compound, 2-methylisoborneol, in a drinking water reservoir. Water Res..

[2] Chorus E.I., Bartram J. (1999). Toxic Cyanobacteria in Water: A Guide to Their Public Health Consequences, Monitoring and Management.

[3] Tabachek J.-A.L., Yurkowski M. (1976). Isolation and identification of blue-green algae producing muddy odor metabolites, geosmin, and 2-methylisoborneol, in saline lakes in manitoba. J. Fish. Board Can..

[4] Izaguirre G., Taylor W. (1995). Geosmin and 2-methylisoborneol production in a major aqueduct system. Water Sci. Technol..

[5] Matsumoto A., Tsuchiya Y. (1988). Earthy-musty odor-producing cyanophytes isolated from five water areas in Tokyo. Water Sci. Technol..

[6] Henley D.E. (1970). Odorous Metabolite and Other Selected Studies of Cyanophyta.

[7] Glaze W.H., Schep R., Chauncey W., Ruth E.C., Zarnoch J.J., Aieta E.M., Tate C.H., McGuire M.J. (1990). Evaluating oxidants for the removal of model taste and odor compounds from a municipal water supply. J. Am. Water Works Assoc..

[8] Dąbrowski A., Podkościelny P., Hubicki Z., Barczak M. (2005). Adsorption of phenolic compounds by activated carbon—A critical review. Chemosphere.

[9] KEITI (2012). Cyanobacterial Bloom Water Management and Respons Technique.

[10] EPA (2015). Recommendations for Public Water Systems to Manage Cyanotoxins in Drinking Water.

[11] Metcalf J.S., Godd G.A. (2014). Cyanobacterial Toxins (Cyanotoxins) in Water.

[12] Zamyadi A., MacLeod S.L., Fan Y., McQuaid N., Dorner S., Sauvé S., Prévost M. (2012). Toxic cyanobacterial breakthrough and accumulation in a drinking water plant: A monitoring and treatment challenge. Water Res..

[13] Kim K.H., Lim B.-J., You K.-A., Park M.-H., Park J.H., Kim B.-H., Hwang S.J. (2014). Identification and analysis of geosmin production potential of anabaena stain isolated from north Han river using genetic methods. Korean J. Environ. Ecol..

[14] Moustafa A., Loram J.E., Hackett J.D., Anderson D.M., Plumley F.G., Bhattacharya D. (2009). Origin of saxitoxin biosynthetic genes in cyanobacteria. PLoS ONE.

[15] Tamura K., Nei M. (1993). Estimation of the number of nucleotide substitutions in the control region of mitochondrial DNA in humans and chimpanzees. Mol. Biol. Evol..

[16] Tsao H.-W., Michinaka A., Yen H.-K., Giglio S., Hobson P., Monis P., Lin T.-F. (2014). Monitoring of geosmin producing anabaena circinalis using quantitative PCR. Water Res..

[17] Ueno Y., Nagata S., Tsutsumi T., Hasegawa A., Watanabe M.F., Park H.-D., Chen G.-C., Chen G., Yu S.-Z. (1996). Detection of microcystins, a blue-green algal hepatotoxin, in drinking water sampled in haimen and fusui, endemic areas of primary liver cancer in China, by highly sensitive immunoassay. Carcinogenesis.

[18] Vaitomaa J., Rantala A., Halinen K., Rouhiainen L., Tallberg P., Mokelke L., Sivonen K. (2003). Quantitative real-time pcr for determination of microcystin synthetase e copy numbers for microcystis and anabaena in lakes. Appl. Environ. Microbiol..

[19] Marchesi J.R., Sato T., Weightman A.J., Martin T.A., Fry J.C., Hiom S.J., Wade W.G. (1998). Design and evaluation of useful bacterium-specific pcr primers that amplify genes coding for bacterial 16s RRNA. Appl. Environ. Microbiol..

[20] Lyra C., Suomalainen S., Gugger M., Vezie C., Sundman P., Paulin L., Sivonen K. (2001). Molecular characterization of planktic cyanobacteria of anabaena, aphanizomenon, microcystis and planktothrix genera. Int. J. Syst. Evolut. Microbiol..

[21] Guillard R.R. (1973). Methods for microflagellates and nannoplankton. Handbook of Phycological Methods: Culture Methods and Growth Measurements.

[22] Stanier R., Kunisawa R., Mandel M., Cohen-Bazire G. (1971). Purification and properties of unicellular blue-green algae (order chroococcales). Bacteriol. Rev..

[23] Nübel U., Garcia-Pichel F., Muyzer G. (1997). Pcr primers to amplify 16s RRNA genes from cyanobacteria. Appl. Environ. Microbiol..

[24] Kim K.H., Yoon Y.D., Hwang S.-J. (2018). Development of molecular probes for evaluating 2-methylisoborneol (2-MIB) production potential in cyanobacterial communities. J. Microbiol..

[25] Oh K.-H., Han A.-W., Cho Y.-C. (2010). Analysis of sequence diversity of mcya gene involved in microcystin synthesis in Korean reservoirs. Korean J. Microbiol..

[26] Hisbergues M., Christiansen G., Rouhiainen L., Sivonen K., Börner T. (2003). PCR-based identification of microcystin-producing genotypes of different cyanobacterial genera. Arch. Microbiol..

[27] MOE (2013). Standard Methods of Environmental Examination and Inspection Act.

[28] APHA (2017). Standard Methods for the Examination of Water and Wastewater.

[29] Vezie C., Brient L., Sivonen K., Bertru G., Lefeuvre J.-C., Salkinoja-Salonen M. (1998). Variation of microcystin content of cyanobacterial blooms and isolated strains in lake grand-lieu (France). Microb. Ecol..

[30] Mihali T.K., Kellmann R., Neilan B.A. (2009). Characterisation of the paralytic shellfish toxin biosynthesis gene clusters in anabaena circinalis awqc131c and aphanizomenon sp. Nh-5. BMC Biochem..

[31] Sivonen K., Niemelä S., Niemi R., Lepistö L., Luoma T., Räsänen L. (1990). Toxic cyanobacteria (blue-green algae) in Finnish fresh and coastal waters. Hydrobiologia.

[32] Saker M.L., Jungblut A.-D., Neilan B.A., Rawn D.F., Vasconcelos V.M. (2005). Detection of microcystin synthetase genes in health food supplements containing the freshwater cyanobacterium aphanizomenon flos-aquae. Toxicon.

[33] Wood S.A., Rasmussen J.P., Holland P.T., Campbell R., Crowe A.L. (2007). First report of the cyanotoxin anatoxin-a from aphanizomenon issatschenkoi (cyanobacteria). J. Phycol..

[34] Jüttner F., Höflacher B., Wurster K. (1986). Seasonal analysis of volatile organic biogenic substances (VOBS) in freshwater phytoplankton populations dominated by dinobryon, microcystis and aphanizomenon. J. Phycol..

[35] Kotak B.G., Kenefick S.L., Fritz D.L., Rousseaux C.G., Prepas E.E., Hrudey S.E. (1993). Occurrence and toxicological evaluation of cyanobacterial toxins in alberta lakes and farm dugouts. Water Res..

[36] Kotak B.G., Lam A.K.Y., Prepas E.E., Kenefick S.L., Hrudey S.E. (1995). Variability of the hepatotoxin microcystin-lr in hypereutrophic drinking water lakes. J. Phycol..

[37] Park H.D., Watanabe M.F., Harada K.I., Suzuki M., Hayashi H., Okino T. (1993). Seasonal variations of microcystis species and toxic heptapeptide microcystins in lake suwa. Environ. Toxicol..

[38] Choi A.-R., Oh H.-M., Lee J. (2002). Ecological study on the toxic microcystis in the lower nakdong river. Algae.

[39] Brownell A.C. (2014). The Roles of Microcystin and Sulfide in Physiology and Tactic Responses of Pathogenic and Non-Pathogenic Mat-Forming Cyanobacteria. Master’s Thesis.

[40] Su M., Yu J., Zhang J., Chen H., An W., Vogt R.D., Andersen T., Jia D., Wang J., Yang M. (2015). Mib-producing cyanobacteria (planktothrix sp.) in a drinking water reservoir: Distribution and odor producing potential. Water Res..

[41] Mez K., Hanselmann K., Naegeli H., Preisig H.R. (1996). Protein phosphatase-inhibiting activity in cyanobacteria from alpine lakes in Switzerland. Phycologia.

[42] Sabater S., Vilalta E., Gaudes A., Guasch H., Munoz I., Romani A. (2003). Ecological implications of mass growth of benthic cyanobacteria in rivers. Aquat. Microb. Ecol..

[43] Izaguirre G., Taylor W. (2004). A guide to geosmin-and mib-producing cyanobacteria in the United States. Water Sci. Technol..

[44] Oudra B., Loudiki M., Vasconcelos V., Sabour B., Sbiyyaa B., Oufdou K., Mezrioui N. (2002). Detection and quantification of microcystins from cyanobacteria strains isolated from reservoirs and ponds in morocco. Environ. Toxicol..

[45] Park H.D., Watanabe M.F., Harada K.I., Nagai H., Suzuki M., Watanabe M., Hayashi H. (1993). Hepatotoxin (microcystin) and neurotoxin (anatoxin-a) contained in natural blooms and strains of cyanobacteria from Japanese freshwaters. Nat. Toxins.

[46] Halinen K., Jokela J., Fewer D.P., Wahlsten M., Sivonen K. (2007). Direct evidence for production of microcystins by anabaena strains from the baltic sea. Appl. Environ. Microbiol..

[47] Mbedi S., Welker M., Fastner J., Wiedner C. (2005). Variability of the microcystin synthetase gene cluster in the genus planktothrix (oscillatoriales, cyanobacteria). FEMS Microbiol. Lett..

[48] Jüttner F., Watson S.B. (2007). Biochemical and ecological control of geosmin and 2-methylisoborneol in source waters. Appl. Environ. Microbiol..

[49] Almanza V., Parra O., Carlos E.D.M., Baeza C., Beltran J., Figueroa R., Urrutia R. (2016). Occurrence of toxic blooms of microcystis aeruginosa in a central chilean (36° lat. S) urban lake. Rev. Chil. Hist. Nat..

[50] Yang Z., Kong F., Zhang M. (2016). Groundwater contamination by microcystin from toxic cyanobacteria blooms in lake chaohu, china. Environ. Monit. Assess..

[51] Tung S.-C., Lin T.-F., Liu C.-L., Lai S.-D. (2004). The effect of oxidants on 2-mib concentration with the presence of cyanobacteria. Water Sci. Technol..

[52] Komárek J., Zapomělová E. (2007). Planktic morphospecies of the cyanobacterial genus anabaena = subg. Dolichospermum—1. Part: Coiled types. Fottea.

[53] Komárek J., Zapomělová E. (2008). Planktic morphospecies of the cyanobacterial genus anabaena = subg. Dolichospermum–2. Part: Straight types. Fottea.

[54] McGregor G. (2013). Freshwater cyanobacteria of north-eastern Australia: 2. Chroococcales. Phytotaxa.

[55] Otsuka S., Suda S., Li R., Watanabe M., Oyaizu H., Matsumoto S., Watanabe M.M. (1998). 16s RDNA sequences and phylogenetic analyses of microcystis strains with and without phycoerythrin. FEMS Microbiol. Lett..

[56] Beltran E.C., Neilan B.A. (2000). Geographical segregation of the neurotoxin-producing cyanobacterium anabaena circinalis. Appl. Environ. Microbiol..

[57] Mori F., Erata M., Watanabe M.M. (2002). Cryopreservation of cyanobacteria and green algae in the nies-collection. Microbiol. Cult. Collect..

[58] Pearson L., Mihali T., Moffitt M., Kellmann R., Neilan B. (2010). On the chemistry, toxicology and genetics of the cyanobacterial toxins, microcystin, nodularin, saxitoxin and cylindrospermopsin. Mar. Drugs.

[59] Tanabe Y., Sano T., Kasai F., Watanabe M.M. (2009). Recombination, cryptic clades and neutral molecular divergence of the microcystin synthetase (MCY) genes of toxic cyanobacterium microcystis aeruginosa. BMC Evolut. Biol..

[60] Chiu Y.-T., Yen H.-K., Lin T.-F. (2016). An alternative method to quantify 2-mib producing cyanobacteria in drinking water reservoirs: Method development and field applications. Environ. Res..

[61] Ngwa F.F., Madramootoo C.A., Jabaji S. (2014). Comparison of cyanobacterial microcystin synthetase (MCY) e gene transcript levels, mcy e gene copies, and biomass as indicators of microcystin risk under laboratory and field conditions. MicrobiologyOpen.

[62] Zhu P., Zhang B.-F., Wu J.-H., Dang C.-Y., Lv Y.-T., Fan J.-Z., Yan X.-J. (2014). Sensitive and rapid detection of microcystin synthetase e gene (mcyE) by loop-mediated isothermal amplification: A new assay for detecting the potential microcystin-producing microcystis in the aquatic ecosystem. Harmful Algae.

[63] Boopathi T., Ki J.-S. (2014). Impact of environmental factors on the regulation of cyanotoxin production. Toxins.

[64] Dittmann E., Neilan B.A., Erhard M., Von Döhren H., Börner T. (1997). Insertional mutagenesis of a peptide synthetase gene that is responsible for hepatotoxin production in the cyanobacterium microcystis aeruginosa PCC 7806. Mol. Microbiol..

[65] Blevins W., Schrader K., Saadoun I. (1995). Comparative physiology of geosmin production by streptomyces halstedii and anabaena sp.. Water Sci. Technol..

[66] Saadoun I.M., Schrader K.K., Blevins W.T. (2001). Environmental and nutritional factors affecting geosmin synthesis by anabaena sp.. Water Res..

[67] Zhang T., Li L., Song L., Chen W. (2009). Effects of temperature and light on the growth and geosmin production of lyngbya kuetzingii (cyanophyta). J. Appl. Phycol..

[68] Mackey K.R., Paytan A., Caldeira K., Grossman A.R., Moran D., McIlvin M., Saito M.A. (2013). Effect of temperature on photosynthesis and growth in marine synechococcus spp.. Plant Physiol..

[69] You K., Byeon M., Youn S., Hwang S., Rhew D. (2013). Growth characteristics of blue-green algae (*Anabaena spiroides*) causing tastes and odors in the north-Han river, Korea. Korean J. Ecol. Environ..

[70] Dziallas C., Grossart H.-P. (2011). Increasing oxygen radicals and water temperature select for toxic microcystis sp.. PLoS ONE.

[71] Rapala J., Sivonen K., Luukkainen R., Niemelä S.I. (1993). Anatoxin-a concentration inanabaena andaphanizomenon under different environmental conditions and comparison of growth by toxic and non-toxicanabaena-strains—A laboratory study. J. Appl. Phycol..

[72] Jähnichen S., Long B.M., Petzoldt T. (2011). Microcystin production by microcystis aeruginosa: Direct regulation by multiple environmental factors. Harmful Algae.

[73] Park H., Kim H., Lee J., Lee J., Lee H., Park J., Seo J., Youn S., Moon J. (2011). Investigation of criterion on harmful algae alert system using correlation between cell numbers and cellular microcystins content of Korean toxic cyanobacteria. J. Korean Soc. Water Qual..

