# Supplementary material for: Harmful Cyanobacterial Material Production in the North Han River (South Korea): Genetic Potential and Temperature-Dependent Properties
Source: Int J Environ Res Public Health. 2018 Mar 3;15(3):444. doi: 10.3390/ijerph15030444 (PMC5876989; doi:10.3390/ijerph15030444)
Supplement: Supplementary file 1 [file ijerph-15-00444-s001.pdf]

Supplementary Material: Harmful Cyanobacterial Material Production in the North Han River (South Korea): Genetic Potential and Temperature-Dependent Properties

Keonhee Kim, Chaehong Park, Youngdae Yoon and Soon-Jin Hwang *

Table S1. BLAST result of harmful-material-synthesizing genes of cyanobacterial strains isolated from the North-Han River, S. Korea.

Cyanobacteria	Target gene	Description	Max Score	Query Cover	E Value	Identity	Accession
Dolichospermum circinale (ST)	*mcyA*	*Microcystis aeruginosa* UTEX 'LB 2385' mcyA (*mcyA*) gene, partial cds	73.1	44%	3e-09	79%	KF372574.1
Aphanizomenon flos-aquae	*mcyA*	*Microcystis aeruginosa* FCY-26 mcyA (*mcyA*) gene, complete cds	241	51%	7e-60	99%	JQ290083.1
Microcystis aeruginosa	*mcyA*	*Microcystis aeruginosa* FCY-26 mcyA (*mcyA*) gene, complete cds	440	95%	7e-120	98%	JQ290083.1
Oscillatoria limosa	*mib*	*Oscillatoria limosa* LBD 305b MIB synthase gene, partial cds	265	100%	3e-67	96%	HQ630885.1
Dolichospermum circinale (ST)	*gys*	*Anabaena ucrainica* CHAB2155 geosmin synthesis operon, complete sequence	1020	98%	0.0	99%	HQ404997.1
Dolichospermum circinale (CT)	*gys*	*Anabaena ucrainica* CHAB2155 geosmin synthesis operon, complete sequence	1020	98%	0.0	99%	HQ404997.1

Remarks: *mcyA*: microcystin synthesis gene, *mib*: 2-methylisoborneol(MIB) synthesis gene, *gys*: geosmin synthesis gene, ST: Straight type, CT: Coiled type.

© 2018 by the authors; licensee MDPI, Basel, Switzerland. This article is an open access article distributed under the terms and conditions of the Creative Commons by Attribution (CC-BY) license (http://creativecommons.org/licenses/by/4.0/).
